# Proposal for a EN 149 acceptable reprocessing method for FFP2 respirators in times of severe shortage

**DOI:** 10.1186/s13756-020-00744-3

**Published:** 2020-06-17

**Authors:** Andreas F. Widmer, Gilles Richner

**Affiliations:** 1 University of Basel Hospitals, Division of infectious Diseases & Hospital Epidemiology and University of Basel, 4031 Basel, Switzerland; 2 Federal Office for Civil Protection FOCP, Spiez Laboratory, Spiez, Switzerland

**Keywords:** Reprocessing, Respirators, FFP2, Sterilization, Infection control, Personal protection equipment

## Abstract

**Introduction:**

Transmission of SARS-CoV-2 to health care workers (HCW) poses a major burden in the current COVID-19 pandemic. Unprotected exposure to a COVID-19 patient is a key risk factor for HCWs. Transmission mainly occurs by droplet transmission, or by aerosol generating procedures. Respirators such as filtering face piece masks (FFP2), also called respirators, are required to prevent transmission during aerosol generating procedures, as part of the personal protective equipment (PPE) for HCWs. However, many HCW were infected due to lack of PPE, or failure to use them. Therefore, the worldwide shortage of respirators triggered the development of reprocessing used FFP2 respirators or N95 respirators as standard in the US. Our proposal with H_2_O_2_ plasma sterilization for decontamination allows to reprocess FFP2, while they still meet the filtration efficiency required by EN 149. The protocol is simple, uses available resources in hospitals and can be rapidly implemented to decrease the shortage of respirators during this crisis. The goal of the study was the evaluate if respirators can be reprocessed and still fulfill the requirements for filtration efficiency outlined by EN 149.

**Methods:**

Used FFP2 respirators – Model 3 M Aura™ 1862+ − were sterilized using a low temperature process hydrogen peroxide (H_2_O_2_), V-PRO® maX Low Temperature, a FDA (Food and Drug Administration) approved method to decontaminate FFP2 respirators. Decontaminated respirators were further checked for residual peroxide by a single-gas detector for H_2_O_2_. The total inward leakage of the protective respirators was quantitatively tested with 10 test persons in an atmosphere charged with paraffin aerosol according to the European Standard EN 149. The fit factor was calculated as the inverse of the total inward leakage.

**Results:**

Ten new and ten decontaminated FFP2 respirators were tested for filtration efficiency. None of the respirators exceeded the maximum acceptable concentration of peroxide. More than 4000 respirators have been reprocessed so far, at cost of approximately 0.3 Euro/piece.

**Conclusions:**

FFP2 respirators can be safely reprocessed once after decontamination with plasma peroxide sterilization, whereafter they still fulfill EN 149 requirements. This allows to almost double the current number of available FFP2 respirators.

## Introduction

March 11, 2020, the World Health Organization (WHO) declared coronavirus disease (COVID-19) a pandemic [1, 2]. More than 2 million individuals have been infected on all continents. At least a part of COVID-19 patients appear to be infectious 2–3 days prior to present symptoms [3]. Current infection control practices for PPE includes gloves, gowns and surgical respirator as minimum standard of care for treating COVID-19 patients in hospitals. During aerosol generating procedures, wearing FFP2 respirators is recommended by the WHO and many national authorities. However, the demand for respirators largely exceeds available resources. Lacking PPE, many HCWs were exposed to COVID-19 patients without appropriate PPE, and contracted the disease [4, 5].

As the use of respirators increases, while supplies are limited, strategies to extend their use have been proposed. In the US, Emergency Use Authorization (EUA) allows to use today even expired National Institute for Occupational Safety and Health (NIOSH) -approved respirators. The Centers for Disease Control and Prevention (CDC) has also issued guidelines on how to create your own cloth respirator. However, filtration efficiency of these cloth respirators is much lower than that of commercially available respirators [6, 7]. One study claimed for influenza, that respirators “can effectively serve as personal bio aerosol samplers.” [8].

Dry heat, UV light and ethylene oxide have been used to decontaminate respirators, but did not ensure efficiency of filtering after decontamination [9]. The protocol of Nebraska (https://www.nebraskamed.com/sites/default/files/documents/covid-19/n-95-decon-process.pdf) also provides an option for reprocessing. In the US, the FDA finally approved on March 30, 2020, the “Battelle respirator-sterilizing technology” for multiple N95 respirators reprocessing using hydrogen peroxide vapour (Clarus C system, Bioquell, Horsham, PA). The system allows safe reprocessing without losing filtration efficiency (https://www.battelle.org/newsroom/news-details/coronavirus-fda-provides-full-ok-for-battelle-respirator-sterilizing-technology).

However, all protocols have some deficiencies, require a an Ultraviolet C (UVc) Light-Emitting Device in the hospital or are dependent on a commercially available reprocessing company, or are not ready for hospitals within days or weeks of shortage.

We looked for a rapid and inexpensive decontamination process, commonly available in hospitals, relying on available human resources and being safe after reprocessing, according to the requirements of EN 149. This European Standard specifies the minimum requirements for filtering masks to be marketed as FFP2 respiratory protective devices. We therefore tested the reprocessed respirators exactly according to EN 149.

## Methods

### Decontamination

Used FFP2 respirators – Model Aura™ 1862+, − were collected in designated containers, and when full, sent to Central Sterilization (CS). Respirators are stored for 24 h at CS: recent data showed that even at high inocula SARS-CoV-19 do not survive storage at room temperature [10]. Staff of CS, protected with appropriate PPE, checked under the magnifying glass each FFP2 respirator for intact surfaces, debris and visual changes, such as residual lipstick, make- up, and other residuals from humans. Respirators that passed this test were individually packed in bags as recommended by the manufacturer. The respirators were sterilized by vaporized hydrogen peroxide (H_2_O_2_) low temp sterilization using the V-PRO® maX Low Temperature Sterilization System (STERIS 5960 Heisley Road Mentor, OH 44060, USA) with short program (https://www.steris.com/healthcare/products/v-pro-sterilizers/v-pro-max-low-temperature-sterilization-system).

Individually packed respirators were further checked for residual H_2_O_2_ before shipping to central storage. They got an individual purchasing number to ensure that reprocessed respirators can be shipped to defined wards only. Residual H_2_O_2_ was detected after the process with a single-gas detector for H_2_O_2,_ Dräger X-am® 5100 (Drägerwerk AG & Co., Lübeck, Germany).

### Testing according to EN 149 after reprocessing

DIN EN 149 is the European standard for FFP respirators, similar to the American standard N95 (NIOSH-42CFR84). The assessment is based on testing the total inward leakage of masks on 10 test individuals.

New and reprocessed respirators (each *n* = 10) have been tested at Spiez Laboratory, Federal Office for Civil Protection FOCP, Switzerland. The total inward leakage of the protective respirators was quantitatively tested in an atmosphere charged with paraffin aerosol according to EN 149. The aerosol concentration was measured with the Portacount® Pro+ Respirator Fit Tester 8038 (TSI Incorporated, Shoreview, USA) outside and inside the respirator while the test persons were performing a series of tasks (movement) according to EN 149. The total inward leakage is the ratio of outside and inside paraffin aerosol concentrations. The fit factor (FF) is the inverse of the total inward leakage (Table 1).

**Table 1:** Fit factors of masks tested with the test persons, new and reprocessed masks. 200 is the upper detection limit of Portacount*®*

Test Person	Fit Factor	
	New	Reprocessed
1	170	> 200
2	> 200	85
3	> 200	> 200
4	189	108
5	> 200	> 200
6	143	127
7	> 200	> 200
8	> 200	152
9	28	23
10	178	130

The requirements according to standard EN 149:2001 + A1:2009 are met for all the 10 test persons, both with new and reprocessed masks

### Cost for reprocessing

The cost was estimated by applying time for decontamination, but did not include time for collecting and distributing respirators. We calculated 0.3 Euro/reprocessed respirator for human resources not taking into account cost for the sterilization. However, we assume the costs for sterilization, handling and transporting of respirators to be a maximum of 0.2 Euro /respirator.

## Results

Around 10% of the more than 5000 respirators were discarded prior sterilization since residual debris (e.g.lipstick, make-up, visible dirt) was detected. More than 4500 FFP2 respirators have been processed by vaporized H_2_O_2_ low temp sterilization using the V-PRO® maX Low Temperature Sterilization System. The process is routinely validated by the device itself: the device automatically interrupts the sterilization process if the defined parameters are not fulfilled [11]. None of the cycles did interrupt. The sterility assurance level requires for sterilization a 6 log reduction of the spores most resistant to the sterilization technique [12]. SARS-CoV-2 belongs to the enveloped viruses that are highly susceptible to this sterilization technique [12] and are therefore killed by this process. Individually packed FFP2 respirators demonstrated 1.5 ± 0.1 mg/m^3^ hydrogen peroxide immediately after sterilization. Unpacked respirators showed a relatively high concentration of H_2_O_2_ immediately after sterilization (3.6 ± 1.5 mg/ m^3^ H_2_O_2_) but aeriation for 24 h lead to very low values of 0.2 ± 0.1 mg/m^3^ H_2_O_2_, which is much below the maximum allowed concentration of 0.7 mg/ m^3^ H_2_O_2_.

After one single reprocessing cycle, all tested FFP2 respirators fulfilled the requirement of the standard EN 149 for FFP2 (Fit Factor > 13, corresponding to a total inward leakage of 8%) (Table 1). The Portacount® setting for FFP2 testing has a higher detection limit of 200, which was reached 9 times over the 20 measurements. Therefore, an average reduction of filtering efficiency cannot be calculated for all the measurements. When possible, average of 25% was obtained (Table 1).

The cost for reprocessing was estimated to be 0.5 Euro/respirator. However, open sterilization without wraps will save additional money when compared to individually wrapped respirators for sterilization as performed here as the bags designed specifically for plasma sterilization are relatively expensive. If the respirator is worn immediately after opening the bag, this potentially exposes the HCWs to residual H_2_O_2_. Open sterilization without wrap saves money, and eliminates any risk for the HCWs because of natural aeration of the respirators in non-airtight boxes.

## Discussion

Respirators have been designed as single-use devices. Reprocessing generally includes multiple steps such disassembling, cleaning, and if necessary, refurbishing after they have been used. Reprocessing must ensure that the product still meets the same requirements as a new product. As of April 9, 2020, the U.S. Food and Drug Administration (FDA) issued an Emergency Use Authorization (EUA) for the emergency use of vaporized H_2_O_2_ low temperature sterilization with the STERIS V-PRO 1 Plus, maX, and maX2 Low Temperature Sterilization Systems to decontaminate N95 respirators to a maximum of 10 times (https://www.fda.gov/media/136843/download). Aerating respirators before use, or even storage of respirators in non-air-tight containers will eliminate the very low risk for exposure of HCWs to H_2_O_2_. The current situation of severe shortages of respirators requires the next best solution to ensure adequate supply of respirators and other parts of PPE.

Several other methods are available to safely decontaminate used FFP2 or equivalent respirators [9, 13]: The Robert-Koch-Institute – the German health institute – recommends dry heat at 65 °C–70 °C for 30 min (https://www.bmas.de/SharedDocs/Downloads/DE/Thema-Arbeitsschutz/einsatz-schutzrespiratoren-einrichtungen-gesundheitswesen.pdf?__blob=publicationFile, accessed April 8, 2020), but do not prove equivalent filtration efficiency.

Our proposal has several advantages: decontamination is safe by using an FDA-approved sterilization technique that uses low temperature. The temperatures are below 70 °C, a temperature that must be tolerated by a FFP2 respirator during 24 h as required by the standard EN 149. The technique is widely available, and requires less human resources than decontamination with UVc. Decontamination with steam sterilization partly destroys the integrity of respirators as mentioned by Dutch authorities (https://www.rivm.nl/en/documenten/reuse-of-ffp2-masks), decreases filtration efficiency and may not be used unless studies demonstrate the safety of this approach [14].

Some limitations must be mentioned: we only reprocessed the respirators once. Multiple reprocessing cycles appear to be safe up to 10 times based on the FDA approval, but may further impede filtration efficiency. Also, when reprocessing multiple times, the number of reprocessing cycles must be securely tracked and thus be directly written on the device, without altering the efficiency of the respirators. Design of other types of respirator may limit extrapolation of this technique to other types of respirators such as N95.

In conclusion, the proposed method for reprocessing FFP2 respirator may allow to decrease the shortage of FFP2 respirators; the necessary technology is widely available in hospitals and can be rapidly introduced during this crisis at low cost. Most importantly, the reprocessed FFP2 respirators still fulfill the requirements for total inward leakage given by the European standard EN 149 for new FFP2 respirators.

## Supplementary information

**Additional file 1.**

## Data Availability

Data sharing not applicable to this article as no datasets were generated or analyzed during the current study.
