# Supplementary material for: Proposal for a EN 149 acceptable reprocessing method for FFP2 respirators in times of severe shortage
Source: Antimicrob Resist Infect Control. 2020 Jun 17;9:88. doi: 10.1186/s13756-020-00744-3 (PMC7298450; doi:10.1186/s13756-020-00744-3)
Supplement: Supplementary file 1 — Additional file 1. [file 13756_2020_744_MOESM1_ESM.docx]

Reprocessing manual for a FFP2 mask with V-PRO® maX Low Temperature Sterilization

1. Collecting used FFP 2 Masks with a specialized mailbox-type container on wards
2. Delivery to the central sterilization (CS) department in plastic containers meeting requirement for transport of infectious waste (eg.g UN approval for infectious waste of Class 6.2, UN 3291)
3. Store for at least 24 hours before opening the container
4. Visual inspection by staff of Central Sterilization with appropriate personal protecting equipment using a magnifying glass for visual contamination and defects (e.g. holes). Rejection of defective and dirty masks, or contaminated masks (most commonly by residual make-up and lipstick)
5. Film packaging: FFP2 respirators are either individually or sets a 10 to 20 packed in sterile bags suitable for H_2_O_2_ sterilization.
   The packaging is carried out according to the recommendation of the manufacturer .
6. Methodology of preparation:
   1. Low temperature process Hydrogen peroxide (H_2_O_2_)
   2. V-PRO® maX Low Temperature Sterilization
       (STERIS 5960 Heisley Road Mentor, OH 44060, USA)
   3. Short program (Materials without lumen – "28 minute Non Lumen Cycle can process loads up to 50 lbs including cameras, light cords, non-lumened endoscopes and batteries", <https://www.steris.com/healthcare/products/v-pro-sterilizers/v-pro-max-low-temperature-sterilization-system>)
7. Keep masks in a non-airtight container for aeration, or if individually packed, have the healthcare worker open the enveloped 1 hour before use
8. Shipment to central distribution center for stockpiling as individually packed items in boxes to 10-20 masks
9. The material is delivered as a non-sterile product, no double packaging
10. Estimated amount of hands-on time for reprocessing 2 min/mask, for visual inspection, packaging and shipment.
